# New Appliances and Things Medical

**Published:** 1905-07-15

**Authors:** 


### NEW APPLIANCES AND THINGS MEDICAL.

[We shall be glad to receive, at our Office, 28 & 29 Southampton Street, Strand, London, W.C., from the manufacturers, specimens of all new preparations and appliances which may be brought out from time to time.]

### BRAND'S NUTRIENT POWDER (DENCE'S PATENT).

(Brand and Co., Mayfair Works, Vauxhall, London, S.W.)

The powder before us consists of muscle fibre from which the moisture has been removed at a temperature below the coagulation temperature of muscle proteids. As average meat contains from 65 per cent. to 75 per cent. of water, the concentration consequent upon the removal of the moisture from meat is quite apparent. Last week we noticed in this journal a meat powder also deprived of much of the moisture present in the original meat, but in which artificial digestion had been allowed to take place, as a result of which peptones and albumoses were formed. In the powder before us this has not been the case, the proteids being present in an unaltered form and exactly as they exist in ordinary muscle fibre. The powder constitutes a highly concentrated nitrogenous food, and may be used either to reinforce fluid meat preparations deficient in actual proteid material, or alone. The dose is one teaspoonful in beef tea, warm milk, or on bread and butter as a sandwich. It must be remembered that the powder is made from raw meat, and that heating it above 100° F. will diminish its digestibility. The powder given in any of the above ways has a pleasant taste, and is a preparation highly to be recommended.

